# Directed Forgetting and the Production Effect: Assessing Strength and Distinctiveness

**DOI:** 10.1027/1618-3169/a000630

**Published:** 2024-12-11

**Authors:** Jackie Spear, J. Nick Reid, Dominic Guitard, Randall K. Jamieson

**Affiliations:** ^1^ Department of Psychology, University of Manitoba, Winnipeg, Manitob, Canada; ^2^ Department of Psychology, University of Northern British Columbia, Canada; ^3^ School of Psychology, Cardiff University, UK

**Keywords:** production effect, directed forgetting, MINERVA 2

## Abstract

**Abstract:** The item-based directed-forgetting effect is explained as a difference in how strongly people encode remember-cued over forget-cued targets. In contrast, the production effect is typically explained as a difference in the distinctiveness of the memory of produced over unproduced targets. The procedural alignment of the two effects – directing participants to remember or forget, produce or not – coupled with their different theoretical explanations (i.e., strength vs. distinctiveness) presents an opportunity to investigate common versus differential effects of elaborative encoding. This study aims to bridge the gap between these two well-established phenomena by comparing the differences in directed forgetting and the production effect in the context of recognition. Mixed- and pure-list designs were utilized to provide an index of each of these mechanisms in both procedures. Along with a standard production effect and directed forgetting effect in the mixed-list conditions, we found evidence for strength primarily driving results in both procedures. Results are explained using a global matching model of recognition memory, MINERVA 2, by assuming varying levels of encoding strength in relation to task demands. Critically, we obtain the best fit using a strength mechanism over a combined strength and distinctiveness mechanism for our data.


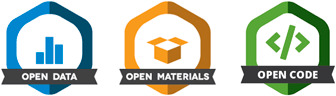


The field of cognitive psychology is filled with numerous demonstrations of robust memory effects that give rise to enhanced performance of one class of items over another (Oberauer et al., 2018). Yet, with many of these demonstrations, much of the field has been working in silos, often lacking consideration of how other related effects could be working under a common theoretical framework. That is, there has been a shortage of attempts to make connections between different memory effects and how they may commonly or differentially influence memory performance.

Directed forgetting and the production effect are two robust cognitive phenomena that have been extensively studied (Hall et al., 2021; MacLeod, 1998; MacLeod et al., 2010; Saint-Aubin et al., 2021). Directed forgetting refers to the ability of individuals to intentionally forget information that is no longer relevant or necessary, while the production effect refers to the improved memory of information that has been actively produced (e.g., spoken aloud or typed) rather than silently read. Some have examined the connection between the two (e.g., Hourihan & MacLeod, 2008), but the effort toward that end has been limited. To build upon the work of Hourihan and MacLeod, we aim to explore the relationship between the two effects through a comprehensive and in-depth investigation.

In a typical item-method directed forgetting procedure, there are two types of items presented at study: remember-cued (R-cued) and forget-cued (F-cued) items. On both types of trials, participants are typically presented with the target word for some duration, which is then followed by the cue to remember (R) or forget (F; see Allen & Vokey, 1998 for an example of simultaneous item and cue presentations). The participants are (falsely) told that items cued to be forgotten will not be tested. A directed forgetting effect is observed when an individual remembers items that they are instructed to remember better than items that they are instructed to forget.

Traditionally, it has been argued that item-method directed forgetting arises from strength, which gives rise to the better recognition of R-cued items over F-cued items. In particular, the selective rehearsal account (Brasden et al., 1993) posits that while the item is presented, the participant engages in maintenance rehearsal to hold the item in working memory while they await the instructional cue. If a cue to remember is then presented, it is posited that participants engage in elaborative rehearsal of the item, whereas if an F-cue is presented, it is posited that participants terminate rehearsal. Therefore, the directed forgetting effect is not due to forgetting per se but rather the strengthening of the memory trace for R-cued items relative to F-cued items due to the additional elaborative encoding. Although most accounts of directed forgetting agree that there is an encoding advantage for R-cued items, it should be noted that other mechanisms have been proposed as well, such as contextual change, selective search, selective rehearsal, retrieval inhibition, and attentional inhibition (Bjork et al., 1968; Epstein, 1969a, 1969b; Fawcett & Taylor, 2008; Hourihan & Taylor, 2006; Montagliani & Hockley, 2019; Sahakyan & Kelley, 2002; Tan et al., 2020; Weiner, 1968; Zacks et al., 1996). Of these accounts, three have been championed: selective rehearsal, retrieval inhibition, and contextual change.

Retrieval inhibition suggests that different mechanisms underlie the list method and item method of directed forgetting. This account proposes that forgetting occurs during the process of retrieval. After the presentation of an F-cue, the items associated with the F-cue are actively inhibited or suppressed. This suppression frees up cognitive resources, allowing more attention and processing to be dedicated to the to-be-remembered items (Bjork, 1989; Brasden et al., 1993).

The contextual change account posits that directed forgetting results from a shift in internal context following an F-cue, as compared to an R-cue. This internal context can refer to mood, emotional state, or even the physical environment (Fawcett et al., 2024; Sahakyan & Kelley, 2002). In practice, the instruction to forget serves as a signal to enter a new internal context. If the original mental state associated with to-be-remembered items is reinstated before retrieval, this context shift can facilitate memory retrieval. By matching the retrieval context to the remembered items, recall is enhanced for R-cued items. The contextual change account is one of the dominant explanations of list-method directed forgetting, where the instructional cue follows an entire list of items rather than each individual item. Recent studies suggest that context change, or context unbinding, may play a role in item-method directed forgetting as well (Chiu et al., 2021; Whitlock et al., 2022), although these studies do not discount the selective rehearsal of R-cued items.

Selective rehearsal suggests that participants intentionally focus on rehearsing the to-be-remembered items while ignoring the to-be-forgotten items (Hourihan & Taylor, 2006; MacLeod, 1998; Woodward & Bjork, 1971). This is done by actively rehearsing R-cued items. Unlike retrieval inhibition, selective rehearsal views forgetting as a more passive process, where unrehearsed F-cued items are simply neglected. Although selective rehearsal is typically associated with the item method, it has been argued that it can offer a unified theory explaining all directed forgetting effects (Sheard & MacLeod, 2005).

In a typical production effect procedure, participants are presented with a list of words, some of which they are instructed to produce (e.g., speak aloud, type, etc.), and some of which they are instructed to read silently. A production effect is observed when participants have better memory for words that they produce than for words that they read silently. The production effect has been examined using multiple modalities, including speaking aloud (Conway & Gathercole, 1987; Hopkins & Edwards, 1972; MacLeod et al., 2010; Murray, 1965a), typing (Jamieson & Spear, 2014), mouthing (Forrin et al., 2012), and even imagining (Jamieson & Spear, 2014). In addition, the phenomenon has been observed across a wide range of paradigms, such as immediate recall, reconstruction of order, free recall, and recognition (Cyr et al., 2022; Gionet et al., 2022; MacLeod et al., 2010; Saint-Aubin et al., 2021). The standard account that has been used to explain the advantage for produced items is the distinctiveness account (but see Bodner et al., 2014, 2020; Taikh & Bodner, 2016). That is, produced items are distinct against a backdrop of nondistinctive read items, where it is the active engagement with produced items that are responsible for the memorial benefit. Therefore, although directed forgetting and the production effect both feature one class of items being better remembered than another due to differential engagement with the items, the accounts for the two effects differ, with directed forgetting being attributed to differences in memory strength and the production effect being attributed to differences in distinctiveness.

With these standard explanations in hand, one possibility is that these two effects, directed forgetting and the production effect, indeed arise for different reasons. However, another possibility is that they arise for similar reasons. If they do arise for the same reasons, the two effects could be explained in the same framework. If they do not arise for the same reasons, then different theoretical frameworks may be needed. Hence, an investigation of the issue is warranted.

Although investigations of directed forgetting and the production effect together have been scarce in the literature, they are not absent. Hourihan and MacLeod (2008) examined the production effect and directed forgetting together, where common versus differential effects on the two procedures were investigated. The two effects were examined using a 2 (produced vs. read) × 2 (remember vs. forget) design to examine the role of directed forgetting when words were produced or when they were read. The study revealed a directed forgetting effect for words that were read but not for those that were produced. These findings suggest that the benefit of production is robust against instructions to forget, lending support to a distinctiveness account.

The current paper aims to conduct a similar investigation to what Hourihan and MacLeod (2008) did, however, in a slightly different way. Instead of examining the production effect and directed forgetting within-subjects, we chose to examine the two effects in a between-subjects design to assess the contributions of strength and/or distinctiveness in each procedure alone. Moreover, assessing both procedures between-subjects offers a clear test of a formal model to assess similarities and differences between the two procedures, which will be illuminated in the work that follows.

To disentangle the two accounts of strength and distinctiveness, often mixed- and pure-list examinations have been utilized (Bodner et al., 2016; Zhou & MacLeod, 2021). In a mixed-list design, sometimes termed a within-subjects production effect, participants encounter a combination of produced and read items. In contrast, in a pure-list design, commonly known as a between-subjects production effect, all items are either produced or read, with no mix of presentation methods. The size of the production effect observed in the mixed- versus the pure-list design is used as an index of the amount of distinctiveness and/or strength that is contributing to the production effect. The signature of a strength effect is an equal benefit for produced items across mixed- and pure-list designs, whereas the signature of a distinctiveness effect is a larger benefit for produced items in a mixed-list design than in a pure-list design.

Given that a distinctiveness effect is observed in a mixed-list design versus a pure-list design, we will additionally conduct a pure-list counterpart. Moreover, to complete the full design, we will also run the pure-list counterpart for directed forgetting. If both effects are strength-based, it will be observed that the benefit for produced or R-cued items is equal across both mixed- and pure-list designs. However, if both effects are distinctiveness-based, it will be observed that there is a larger benefit for produced/R-cued items in the mixed-list designs than in the pure-list designs. Thus, if both effects arise for the same reasons, we expect that the results across both paradigms will be consistent with either a strength- or distinctiveness-based account. If they arise for different reasons, consistent with previous work, we should observe distinctiveness-based results in the production effect (larger effect for mixed-lists than pure-lists) and strength-based results in directed forgetting (similar-sized effects in mixed and pure lists).

Furthermore, in terms of the pattern of findings with the production effect in mixed-list designs and other associated effects (e.g., the generation effect), it has been unclear whether it is a cost to unproduced items that drives the production effect or if it is a benefit to produced items (Begg & Snider, 1987; MacLeod et al., 2010). To answer the question, often the approach is again to run a pure-list counterpart and then compare the hit rates for read and produced items between the two designs. Evidence in favor of a benefit to produced items is acquired if the hit rate for the produced items is larger in the mixed-list design than in the pure-list design. Conversely, evidence in favor of a cost to unproduced items (i.e., the lazy reading hypothesis; Bodner et al., 2014) is acquired if the hit rate for read items is lower in the mixed-list design than in the pure-list design. Bodner et al. (2014) employed these mixed- and pure-list conditions and found that the hit rate for read items was lower in the mixed-list design than in the pure-list design, suggesting that the production effect is driven by a cost to unproduced items. Additionally, the meta-analysis conducted by Bodner et al. showed that there was a cost for silent items with little benefit for produced items. Forrin et al. (2016) similarly found a larger production effect in mixed lists than in pure lists. However, it is unclear whether these patterns of findings hold when other modalities of production are used (e.g., typing).

To date, there have been a few approaches to modeling the production effect, including REM, the Revised Feature Model (RFM), attentional subsetting theory (AST), and MINERVA 2 (Caplan & Guitard, 2024; Jamieson et al., 2016; Kelly et al., 2022; Saint-Aubin et al., 2021). Common to all of these models is the addition of features to a memory trace to account for the added benefit of production. Additionally, directed forgetting has been accounted for using a strength-based version of MINERVA 2 (Reid & Jamieson, 2022; Reid et al., 2023). Thus, the current paper utilizes the MINERVA 2 model, as this model has been used to successfully model the production effect and directed forgetting.

In the modeling framework of the production effect proposed by Jamieson et al. (2016), enhanced memory performance for produced items can be accounted for in one of two ways. In a strength-based account, it is assumed that produced items are better encoded into memory, with more intact features. In a distinctiveness-based account, it is assumed that produced items are elaboratively encoded, such that these items are encoded with more unique, distinct features to distinguish themselves from unproduced or nonelaboratively encoded items. In practice, the model affords memory a global familiarity signal, whereby the system is reminded of the act of production with an iterative retrieval function.

Thus, a theoretical question is: do the parallel explanations for strength versus distinctiveness in directed forgetting and the production effect necessitate their complete independence, or do they potentially operate in tandem, with certain circumstances favoring one or the other? Moreover, can results from both the production effect and directed forgetting, two effects of elaborative processing, be accounted for in a single model? Having a comprehensive model that can account for multiple memory effects can help us escape a siloed approach to memory research and instead consider in tandem different memory effects. To answer the question, we conducted experiments where both mixed and pure lists were used across both the production effect and directed forgetting.

## Experiment 1

**Figure 1:**
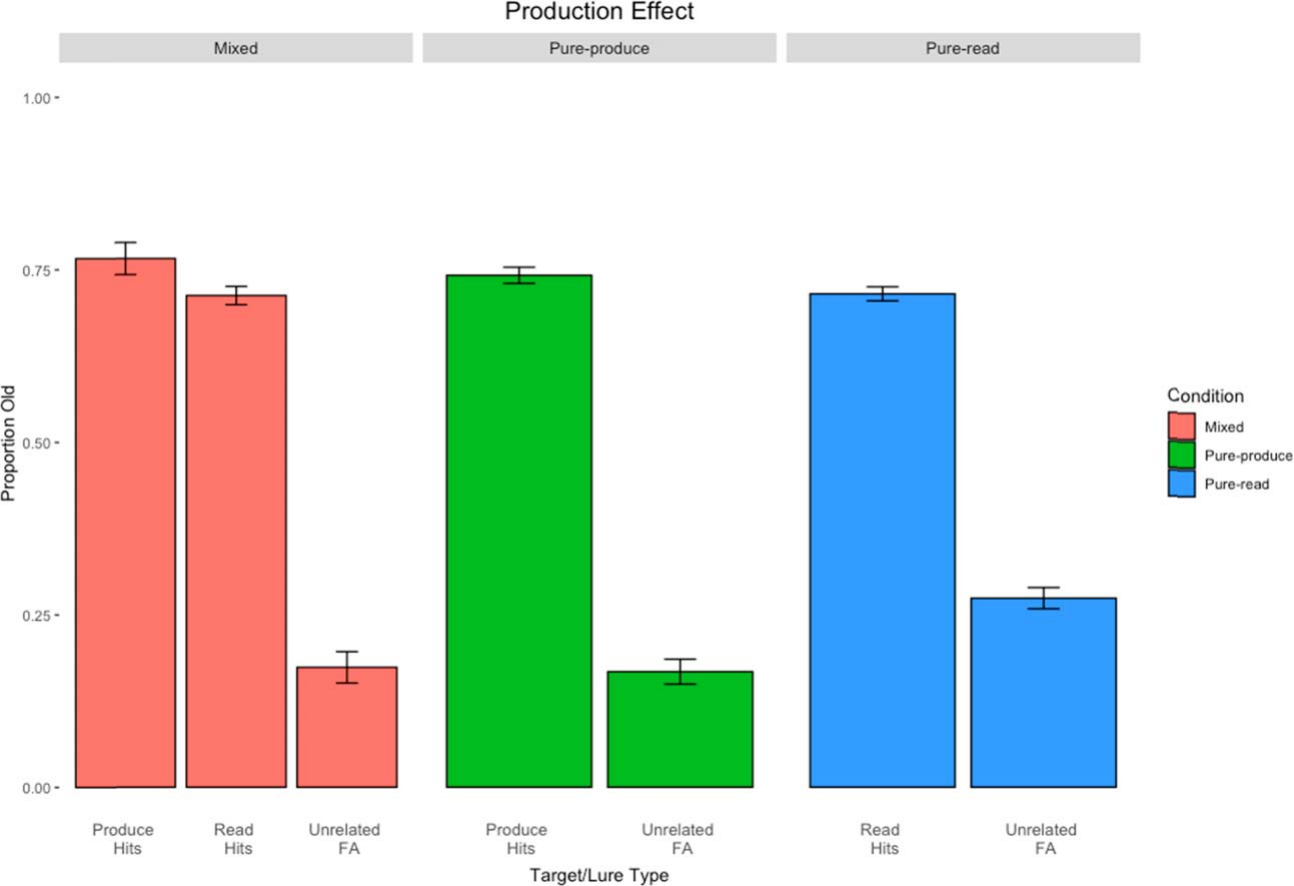
Results from Experiment 1. Error bars represent standard errors of the means.

The goal of Experiment 1 was to examine the production effect in both mixed- and pure-lists using uncategorized words. The modality of production was a production-by-typing task (Bodner et al., 2016; Forrin et al., 2012; Jamieson & Spear, 2014; Kelly et al., 2024). We chose a production-by-typing procedure as this modality is both understudied and provides a convenient way to collect data online. However, more importantly, it is unclear whether the effect should be larger in mixed- relative to pure-lists, as the features encoded in this task can be considered less rich than in spoken production. The standard design was adopted to provide a clean test of the model and to subsequently compare the results from directed forgetting in Experiment 2. Moreover, the inclusion of both mixed- and pure-lists allowed for the assessment of strength and/or distinctiveness mechanisms. Results are displayed in Figure 1.

### Method

#### Participants

According to a two-tailed 80% power analysis conducted with G*Power 3.1 (Faul et al., 2009), a minimum of 15 participants were needed, with alpha set to 0.05. We used an effect size of *d* = 0.81, obtained from Jamieson and Spear (2014). However, as the current design involved three different conditions (a mixed-list, pure-produce, and a pure-read condition) we sought to exceed this amount by more than threefold, as we included an additional procedural change of collecting our data online. As such, data was collected from a total of 128 participants. From there, data from 120 participants (71 female, 49 male) were included in the analysis. Participants were recruited online from the University of Manitoba SONA psychology participant pool and received one credit towards completion of their “Introduction to Psychology” course. The mean age of participants was 20.75 years (range = 17–44, *SD* = 4.9 years). Participants were excluded based on not complying with the instruction manipulation in the production condition (e.g., not typing at least 80% of the time on produce trials or typing on read trials) or if they reported doing something else during the experiment (e.g., some participants reported that they were in class, watching TV, etc.). Of the eight participants who were excluded, four self-reported that they were distracted while doing the experiment, and four participants did not comply with the experimental instructions at least 80% of the time. Data was collected to ensure that there were at least 40 participants in each of the three conditions (the mixed, pure-produce, and pure-read conditions).

#### Materials

Materials were 120 words taken from MacDonald and MacLeod (1998) and are listed in the Appendix. From these 120 words, 80 words were randomly selected for each participant: 20 to serve as produced targets, 20 to serve as read targets, and 40 to serve as new, unstudied lures. In addition to the 40 study words, there were two buffer items at the beginning and end of the study list to mitigate contamination from primacy and recency effects on performance.

#### Procedure

Participants were tested online using jsPsych version 6.3.1, a JavaScript library for running behavioral experiments via a web browser (https://jspsych.org; de Leeuw, 2015). The experiment was hosted online using GitHub Pages. When the study commenced, participants were randomly assigned to a mixed, pure-produce, or pure-read condition.

All participants provided informed consent, which was followed by instructions. The instructions asked participants to turn off any background audio and to remain in full screen for the duration of the experiment. After this, they were presented with instructions for the experiment. The production task involved typing the word that was presented. Participants were instructed to type words that appeared in green and to silently read words that appeared in red. Also, embedded within these instructions was an attention check. Participants were told that when asked to “provide an answer” on the following screen, they should provide the answer to “2 + 2 = __.” If the participant typed in anything but the number “4,” the experiment looped back to the instruction screen until the correct answer was provided.

Before the start of the main experiment, participants were given a short practice phase that included “produce” and “read” trials that provided feedback on their performance. The practice study phase consisted of 16 trials, eight of which were “produce” trials, and eight of which were “read” trials, presented in random order. For the “produce” trials, if participants typed the word correctly, they were presented with feedback that read, “Correct! You typed correctly!.” If participants typed anything except the exact word, they were presented with feedback that read, “Incorrect. You must type the exact word.” For the “read” trials, if participants did not type anything, they were presented with feedback that read, “Correct! Thank you for reading!.” Conversely, if the participant typed anything on “read” trials, they were presented with feedback that read “Incorrect. Please do not type.” This was followed by a practice test phase in which participants also received feedback on their recognition performance.

Once the study phase commenced, study words were first presented in black for 1,000 ms, after which each word immediately turned red or green and remained on the screen for 3,000 ms. Each study word appeared one at a time, in easy-to-read 48px font on a white background. After each study word, the screen was cleared for 500 ms, after which the next study word appeared. Other than the two buffer items at the beginning and end of each study list, all 40 study items were presented in random order.

After the study phase was over, participants were presented with test instructions, which informed them to press ‘y’ if they recognized a word and ‘n’ if they did not. During the test phase, participants were tested on their recognition of all previously studied words (other than the buffer items), along with an equal number of unstudied lures. Each test word appeared in the center of the screen, in black 48 px font, on a white background, until the participant pressed ‘y’ or ‘n’. Once the participant responded, the screen was cleared for 500 ms, after which the next test word appeared. This procedure was repeated until all 80 test words were presented in random order. After the test phase was finished, participants were presented with a demographic questionnaire, followed by a question asking if they were doing anything else while completing the experiment, and finally, a debriefing screen.

### Results and Discussion

All analyses were conducted in R (R Development Core Team, 2022; version 4.2.1, 2022). The results of the mixed-list (leftmost column), pure-produce (middle column), and pure-read (rightmost column) conditions are displayed in Figure 1.

We began with an analysis of the mixed-list results. The first comparison revealed that participants responded “yes” significantly more to old items than new ones, *t*(39) = 19.26, *p* < .001, *d* = 0.98. Next, the comparison between produced versus read items revealed a significant production effect, *t*(39) = 2.14, *p* = .04, *d* = 0.09, with a production advantage of 6%. The pure-list production effect was not significant using the Welch correction for unequal variances, *t*(74.92) = 0.86, *p* = .39, *d* = 0.19, with a production advantage of only 2%.

#### Strength or Distinctiveness?

If it is strength driving the results in the production effect, then the size of the production effect in the mixed- and pure-list conditions should be of similar size. However, if it is primarily due to distinctiveness, then the size of the production effect ought to be significantly larger in the mixed-list condition compared to the pure-list condition. To assess the role of distinctiveness in the mixed-list condition, we next applied an Erlebacher analysis.

The current examination involved three different between-subjects conditions: a mixed-list condition, a pure-produce condition, and a pure-read condition. Production (produced vs. read) in the mixed-list is a within-subjects condition, whereas it is a between-subjects manipulation across the two pure-list conditions. As such, to properly assess the difference in magnitude of the mixed-list and pure-list production effects, the Erlebacher method of analysis was used, a technique that circumvents traditional methods (Erlebacher, 1977). This method ensures an unbiased estimate of the design type and its interaction with the independent variable. R code that was developed by Merritt et al. (2014) was used for this analysis.

A two (item type: produced vs. read) × 2 (design: mixed vs. pure) Erlebacher ANOVA was conducted on only hits. Although there was only a 4% difference between the mixed- versus pure-list production effects, the results revealed a main effect of production, *F*(1, 78) = 4.05, *p* < .05, η^2^ = 0.02. In contrast, there was no main effect of design type, *F*(1, 78) = 0.14, *p* = .71, η^2^ = 0.001, nor an interaction between design type and item type, *F*(1, 78) = 0.45, *p* = .50, η^2^ = 0.002. Thus, the mixed-list production effect was not significantly larger than the pure-list counterpart, failing to lend support to a distinctiveness account.

#### A Cost or a Benefit?

Lastly, in terms of costs and benefits of production, it is assumed that a benefit to produced items is observed if the hit rate for produced items in the mixed-list condition is larger than in the pure-produce condition. Conversely, it is assumed that there is a cost to unproduced items if the hit rate for read items in the mixed-list condition is lower than the hit rate for read items in the pure-read condition.

The hit rate for produced items in the mixed-list condition was 77%, with a 74% hit rate in the pure-produce counterpart (a 3% benefit for the mixed-list). Conversely, the hit rate for read items in the mixed list versus the pure-read list only differed by 1% (71% vs. 72%, respectively). Thus, results from Experiment 1 yielded support for a slight benefit of production, although not a strong one.

## Experiment 2

The purpose of Experiment 2 was to assess the directed forgetting procedure using the same materials and design as before. This consistency allowed for a direct comparison of the production and directed forgetting effects. Both mixed- and pure-lists were utilized to evaluate the roles of strength and distinctiveness in directed forgetting. The standard design of presenting the cue to remember or forget after item presentation was once again adopted to ensure a clear test of the model.

### Method

#### Participants

Participants were recruited in the same way as in Experiment 1. Data was collected from a total of 131 participants. From there, data from 120 participants (71 female, 49 male) were included in the analysis. Eleven participants were excluded based on self-reporting that they were distracted while completing the experiment. The mean age of participants was 20.50 years (range = 17–43, *SD* = 3.8 years). Data was collected to ensure that there were 40 usable participants in each of the mixed, pure-remember, and pure-forget conditions.

#### Materials

The materials were the same as in Experiment 1.

#### Procedure

The procedure was identical to Experiment 1, with the difference that participants were instructed to remember the words presented in green and forget the words presented in red (no typing instruction was given). Moreover, no typing feedback was provided during the practice phase, as no typing instruction was given for the directed forgetting procedure. As before, participants were randomly assigned to one of the three between-subjects conditions: mixed, pure-remember, or pure-read. Participants were not explicitly informed of what condition they were assigned to. Lastly, the experimental test instructions in the directed forgetting procedure (presented after the study phase) additionally included a sentence that instructed participants to identify any items they remembered as old, regardless of the remember and forget cues.

### Results and Discussion

**Figure 2:**
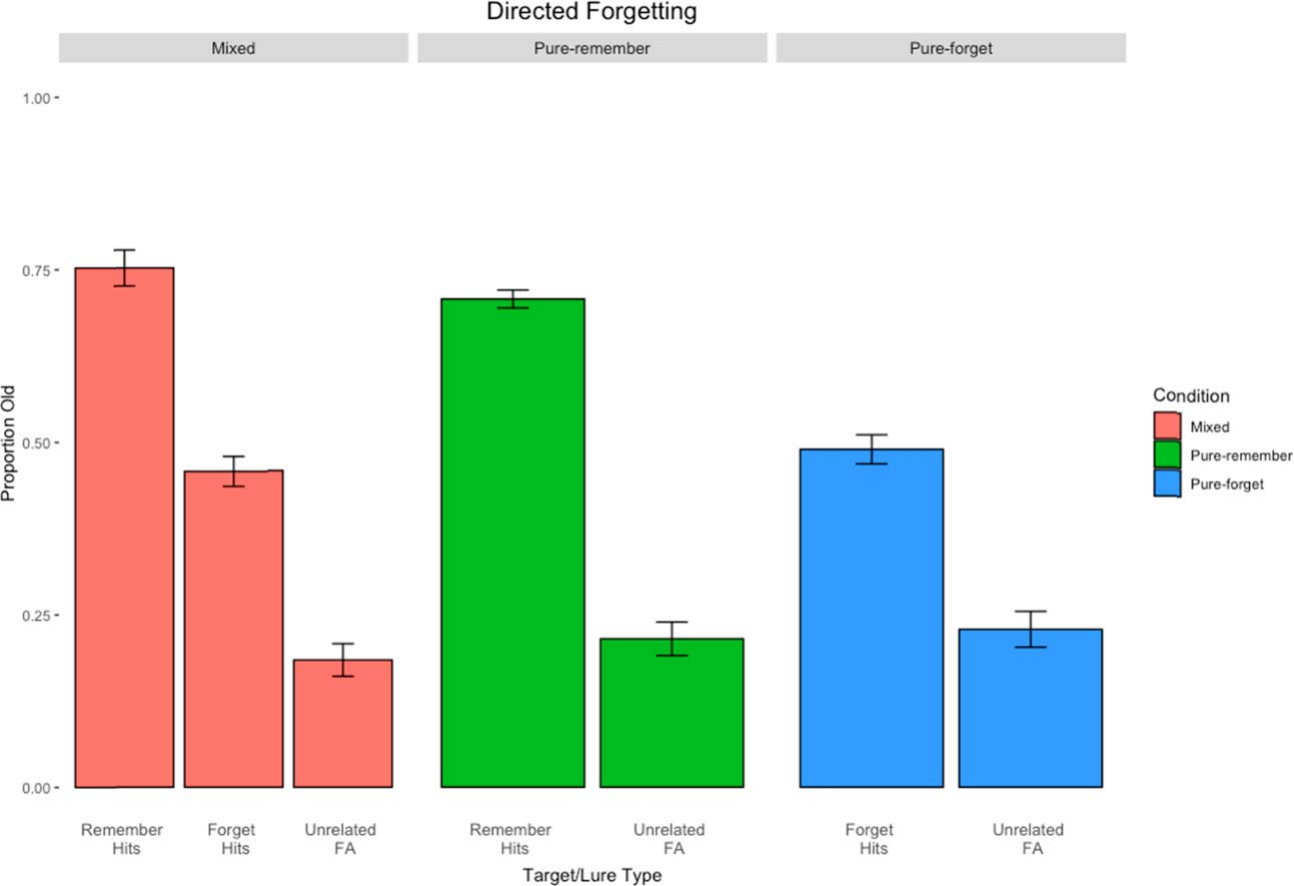
Results from Experiment 2. Error bars represent standard errors of the means.

Figure 2 displays the directed forgetting results of the mixed-list condition in the first column, the pure-produce condition in the second column, and the pure-read condition in the third column. Proportion old is on the *y*-axis, and item-type is on the *x*-axis.

The first comparison revealed that, unsurprisingly, participants responded *yes* to items that they studied significantly more than to items that they did not (i.e., old vs. new items), *t*(39) = 15.16, *p* < .001, *d* = 072. More critically, a mixed-list directed forgetting effect was observed, *t*(39) = 6.71, *p* < .001, *d* = 0.45, with an R-cue advantage of 29%. A pure-list directed forgetting effect was also observed using the Welch correction for unequal variances, *t*(63.63) = 4.69, *p* < .001, *d* = 1.05, with a 22% advantage for R-cued items.

#### Strength or Distinctiveness?

Although there was a 7% difference between the mixed- versus pure-list directed forgetting effects, the two (item type: produced vs. read) × 2 (design: mixed vs. pure) Erlebacher ANOVA conducted on only hits revealed a main effect of cue type, *F*(1, 78) = 64.19, *p* < .001, η^2^ = 0.26, but no main effect of design type, *F(*1, 78) = 0.04, *p* = .84, η^2^ < 0.001, nor an interaction between design type and item type, *F*(1, 78) = 1.39, *p* = .24, η^2^ = 0.006. As in Experiment 1, the mixed-list directed forgetting effect was not significantly larger than the pure-list counterpart, again failing to support a distinctiveness account in directed forgetting.

#### A Cost or a Benefit?

As in Experiment 1, to assess whether there is a cost to F-cued items or a benefit to R-cued items, a comparison of the hit rates for each item type can be done across the mixed- and pure-list conditions.

Experiment 2 yielded mixed results. The hit rate for R-cued items in the mixed-list condition was 75%, whereas the hit rate for the pure-remember condition was 71% (a 4% benefit for the mixed list). However, the hit rate for F-cued items in the mixed list was 46%, whereas it was 49% in the pure-forget condition, indicating a 3% difference in favor of the pure-forget condition. Thus, it appears there was a benefit (higher hit rate for R-cued items in the mixed- than pure-remember condition) and a cost (lower hit rate for F-cued items in the mixed- than pure-forget condition) associated with R-cued and F-cued items in the directed forgetting procedure, of nearly the same magnitude.

#### Experiment 1 and Experiment 2

As there was a larger directed forgetting effect than a production effect, an analysis of the two types of targets across the two procedures in the mixed list was conducted. A mixed ANOVA with item type (produced vs. read) as the within-subjects factor and experiment (directed forgetting vs. production effect) as the between-subjects factor was used to assess this difference. The analysis confirmed that this difference was statistically significant, with a main effect of experiment *F*(1, 78) = 13.35, *p* < .001, η_*p*_^2^ = 0.15. There was also a main effect of item type *F*(1, 78) = 47.48, *p* < .001, η_*p*_^2^ = 0.38, and a significant interaction between Experiment and item type *F*(1, 78) = 22.72, *p* < .001, η_*p*_^2^ = 0.23, indicating that the elaborative encoding benefit (or cost) in directed forgetting (29%) exceeded that of the production effect procedure (6%).

## Experiment 3

Given that the cue to produce or read in a production effect procedure is typically presented concurrently with stimulus presentation, it could be argued that our production effect procedure in Experiment 1 deviated from the standard methodology. Therefore, we conducted Experiment 3 to examine the production effect in a conventional manner. Additionally, we sought to explore any differences between presenting the instruction to produce or read concurrently with the stimulus versus presenting it shortly thereafter, as was done in Experiment 1.

### Method

#### Participants

Participants were recruited in the same way as in Experiment 1. Data was collected from a total of 44 participants. From there, data from 40 participants (25 female and 15 male) were included in the analysis. Four participants were excluded based on self-reporting that they were distracted while completing the experiment. The mean age of participants was 25.33 years (range = 17–47, *SD* = 7.9 years).

#### Materials

The materials were the same as in Experiment 1.

#### Procedure

**Figure 3:**
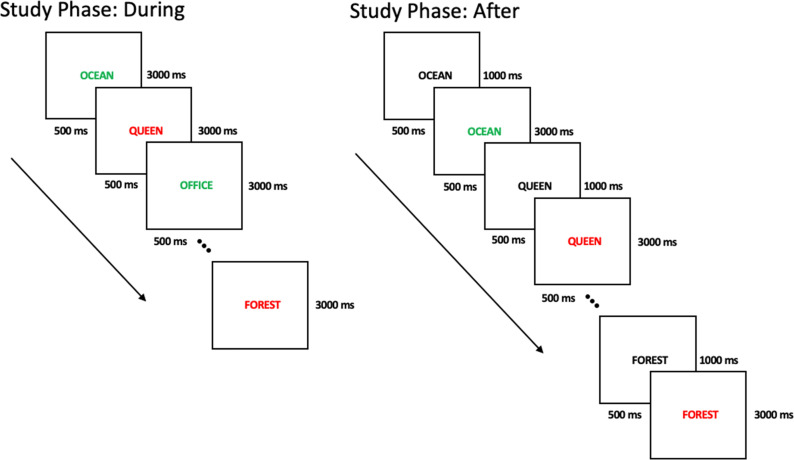
An example of the two study procedures. The *after* study procedure was used in Experiments 1 and 2, and the *during* study procedure was used in Experiment 3. The duration of stimulus presentation is displayed on the right, whereas ISI is displayed on the left.

The procedure was identical to that of Experiment 1, except that the instruction to *produce* or *read* was presented concurrently with the stimulus. Figure 3 illustrates both procedures, with the concurrent stimulus presentation procedure shown on the left-hand side.

### Results and Discussion

**Figure 4:**
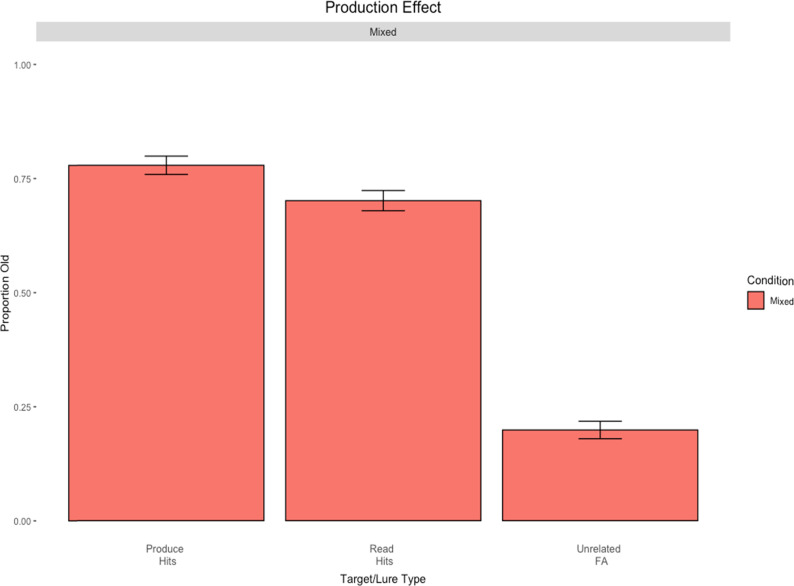
Results from Experiment 3. Error bars represent standard errors of the means.

Figure 4 displays the results of the mixed-list production effect procedure when the cue to produce or read was presented concurrently with the stimulus presentation.

Analyses revealed that participants responded *yes* significantly more to old items than new, *t*(39) = 15.88, *p* < .001, *d* = 0.93. More importantly, there was a significant production effect, *t*(39) = 3.46, *p* = .001, *d* = 0.14, with a production advantage of 8%.

Since the purpose of Experiment 3 was solely to assess the difference between cue presentations (during vs. after instruction presentation), the pure-list counterpart was not conducted. However, to determine if the size of the production effect differed between the two cue presentation timings, an analysis was performed on the targets from Experiment 1 and Experiment 3. A 2 (cue presentation: during vs. after) × 2 (item type: produce vs. read) ANOVA revealed a main effect of item type, *F*(1, 78) = 15.21, *p* < .001, η_*p*_^2^ = 0.16, but no main effect of cue presentation timing, *F*(1, 78) < 0.01, *p* = .99, η_*p*_^2^ < 0.001, nor an interaction between cue presentation timing and item type, *F*(1, 78) = 0.50, *p* = .48, η_*p*_^2^ = 0.01. Thus, there was no significant difference between presenting the cue to produce or read either concurrently or shortly thereafter.

## Summary

The production effect and directed forgetting effect are two types of elaborative processing study procedures that lend a benefit to one class of items over another. Empirical results thus far indicate that there is a sizable difference in the magnitude of this benefit in favor of directed forgetting across the two procedures. Moreover, whether the cue to produce or read is presented concurrently with stimulus presentation or shortly thereafter does not seem to affect the production benefit in the current examination.

The next major aim of this paper was to account for both the production effect and directed forgetting using a computational model of human memory, MINERVA 2. As such, we implemented the elaborative processing account in MINERVA 2 to model the results of Experiments 1–3.

### The Model

To date, there have been four main approaches to modeling the production effect: REM, the Revised Feature Model (RFM), AST, and MINERVA 2 (Caplan & Guitard, 2024; Jamieson et al., 2016; Kelly et al., 2022; Saint-Aubin et al., 2021). Common to all of these models is the addition of features to account for the added benefit of production. The RFM is an account of recall and not recognition, whereas REM and MINERVA 2 are accounts of recognition. In this paper, we use the MINERVA 2 model of recognition memory, which has also been used to model the directed forgetting effect (Reid et al., 2023; Reid & Jamieson, 2022).

MINERVA 2 (Hintzman, 1984, 1986, 1988) belongs to a class of computational models of human memory known as global matching models. MINERVA 2 accounts for memory storage, retrieval, and decision. The model has had a wide range of successes in several cognitive domains, including reaction time (Jamieson & Mewhort, 2009), false recognition (Arndt & Hirshman, 1998), associative learning (Jamieson et al., 2012), decision-making (Dougherty et al., 1999), sentence memory (Reid & Jamieson, 2023), lexical disambiguation (Jamieson et al., 2018), serial recall (Guitard et al., 2025), and implicit rule learning with semantics (Chubala et al., 2016). The model assumes that memory is a matrix, where each row represents an item and columns represent the features of the items. When words are encoded, they are encoded to a unique row in the memory matrix, with some degree of noise. This noise is introduced by the parameter *L*, which is the learning parameter in the model. *L* can also be considered the strength with which a trace is encoded into memory.

Specific to the production effect, there are a few differences from the standard instantiation of the model. Firstly, the benefit of production to memory can be accounted for in one of two ways: with a distinctiveness-based mechanism or a strength-based mechanism. In both cases, a word is first represented by a unique vector, where some number of base features represents the word.

A distinctiveness-based mechanism works by adding some number of extra features to all items. For produced items, these extra features contain additional information, whereas for unproduced items, the extra features contain no additional information (these features are set to 0). Secondly, the model’s retrieval process works in an iterative fashion, akin to the deblurring process utilized by Hintzman (1986). Functionally, the test word is used as a retrieval probe to retrieve an echo three times. On the first iteration, only the base features are included in the probe. For the second iteration, if the word was produced, the retrieved echo content (which serves as the new probe) will now include some of the information about the extra produced features that were encoded during the study phase, thus retrieving new unique features to account for distinctiveness. For the third iteration, the probe is further refined, incorporating both the base and any additional features associated with production, enhancing the retrieval accuracy. This iterative process increases the likelihood of correctly identifying the produced items due to retrieval and use of the enriched feature set. Conversely, for unproduced items, the probe is similarly submitted to memory, and the same iterative retrieval process is used, but the echoes do not strongly pick up any additional production features since the traces in memory they match most strongly do not include these features. Therefore, the probe remains based primarily on the base features over the retrieval iterations, leading to a less distinct and weaker retrieval signal. After the third iteration, a global familiarity signal known as echo intensity is calculated, which is based on the sum of all activations in memory. Activations are calculated based on the similarity of the probe (echo retrieved after the third iteration) to items in memory. The probes for produced items elicit a stronger familiarity signal because they match their corresponding traces in memory on both base and production features, whereas the unproduced probes match only on base features.

In contrast, in a strength-based model of the production effect, there are no extra features added to memory. Instead, it is assumed that produced items are encoded more strongly in memory than read items by varying the parameter *L* in the model for each class of item. This is the same way that Reid and Jamieson (2022) modeled the item-method directed forgetting effect. By assuming that R-cued items are more strongly encoded with more intact features than F-cued items, Reid and Jamieson were able to demonstrate the typical directed forgetting effect found in veridical recognition, as well as a parallel directed forgetting effect that occurs in false recognition for related lures (see Marche et al., 2005; Montagliani & Hockley, 2019; Reid et al., 2023).

Given that there is a mixture of findings found in the literature and that strength and distinctiveness likely work together in tandem given these findings, we present a model that incorporates both strength and distinctiveness mechanisms. First, to implement strength, as outlined above, we can assume that elaboratively studied items are encoded into memory with more intact features than nonelaborative items, by varying the parameter *L* for each class of item (*L*_*P*_ > *L*_*R*_ and *L*_*R*_ > *L*_*F*_). To implement distinctiveness, we assume that distinctive items (e.g., produced items) have additional non-zero features to the base features, whereas for nondistinctive items, the additional features have values of zero. However, because the items are not produced at test (see Jamieson et al., 2016), it is assumed that the initial probe does not contain the distinctive features, but that these features must be retrieved from memory through an iterative retrieval process. Retrieval works in the following fashion: when a probe is presented to memory, activation is similarity-based and is calculated on a feature-to-feature basis in parallel. These activated traces are represented in an echo, where an echo is made up of two key properties: echo content and echo intensity. Echo content, *c*, is a vector comprised of the sum of all the traces that are activated:

$$c_{j}=\overset{m}{\underset{i=1}{\sum }}a_{i}\times M_{ij}\left\{\text{for each }j=1\ldots n\right\}$$

where *c*_*j*_ is the *j*th element of the echo, *a*_*i*_ is the activation for the *i*th trace in memory, *M*_*ij*_ is the *j*th element of the *i*th trace in memory, *m* is the number of traces in memory, and *n* is the number of elements in each vector. The activation for each trace in memory is computed as the cosine similarity between the probe and that trace raised to the exponent of three (Hintzman, 1986, 1988). The retrieval process works in an iterative fashion, such that the test word is used as a retrieval probe to retrieve an echo three times. On the first iteration, only the base features are included in the probe. On the second iteration, if the word was produced, the retrieved echo content (which serves as the new probe) will now include some of the information about the extra produced features that were encoded during the study phase.

Following three iterations, the probe’s familiarity is computed as an echo intensity, which is the sum of the activation elicited by the probe:

$$f=\overset{i=m}{\underset{i=1}{\sum }}{\left(\frac{\sum_{j=1}^{j=d}p_{j\;}\times M_{ij}}{\sqrt{\sum_{j=1}^{j=d}p_{j}^{2}}\sqrt{\overset{j=d}{\underset{j=1}{\sum }}M_{ij}^{2}}}\right)}^{3}$$

where a familiarity (*f*) value is calculated based on the probe's similarity to all traces in memory, *M*, where specifically, a cosine similarity calculation is used, *p*_*j*_ is feature *j* of the probe, *M*_*ij*_ is feature *j* of trace *i* in memory, *m* is the number of memory traces, and *d* is the dimensionality of these traces. This similarity calculation is then converted to activation by raising it to the exponent of three, enhancing the signal-to-noise ratio of all calculated familiarity values. Finally, all these similarities are summed to yield an overall familiarity index, *f*, also called an echo intensity in other uses of the MINERVA model. The additional nonzero feature encoded at study, along with the iterative retrieval process to retrieve those features, is how distinctiveness is represented and used in the model.

The model simulates decision-making by using all the calculated familiarity values (for both old and new words) to determine a criterion based on a chosen percentile that best fits the data. For example, if the decision criterion is set to the 55^th^ percentile, that would mean that the top 45% of the most familiar echo intensities would be classified as *OLD* (a decision criterion of 0.45), while the remaining echo intensities would be classified as *NEW*. In this example, this value represents a slightly conservative criterion, indicating that the model is marginally more inclined to classify items as NEW, being that the criterion is just above the median.

### Simulation Results

The standard design of the production effect and directed forgetting procedures examined here were used to provide an articulate basis for the model and the comparison of the two procedures. We report simulation results from the production effect findings in Experiments 1 and 3, and the directed forgetting findings in Experiment 2. In each simulation, 1,000 independent simulations were conducted.

#### Word Representations

Classically, word representations in the MINERVA 2 framework have been discrete random representations (e.g., a vector of randomly sampled +1s and −1s). However, MINERVA 2 allows for other types of representations, such as engineered representations (e.g., Arndt & Hirshman, 1998), or those derived from models of natural language processing (see Chang & Johns, 2023; Chubala et al., 2016; Reid & Jamieson, 2023; for demonstrations). Here, we use orthogonal continuous representations drawn from a normal distribution, with *M* = 0 and a σ = 1/√(d), where *d* is the dimensionality of each word vector (see Jamieson & Hauri, 2012; Jones & Mewhort, 2007; Murdock, 1982). In the simulations that follow, *d* was set to 300 for the base features.

### Simulation of Experiment 1

We used a combined version of MINERVA 2, where some additional features were added to account for distinctiveness. The model also assumes that produce targets are encoded more strongly into memory with a higher value of *L* than the words that participants were instructed to read. Familiarity was then computed for all 80 test items: 40 old and 40 new. Then, these familiarity values were converted to an old/new decision by comparing them to a chosen criterion that best fits the data. In the simulation of Experiment 1 and in the simulations that follow, we iteratively fit our data by varying values of *L* and the number of production features.

**Figure 5:**
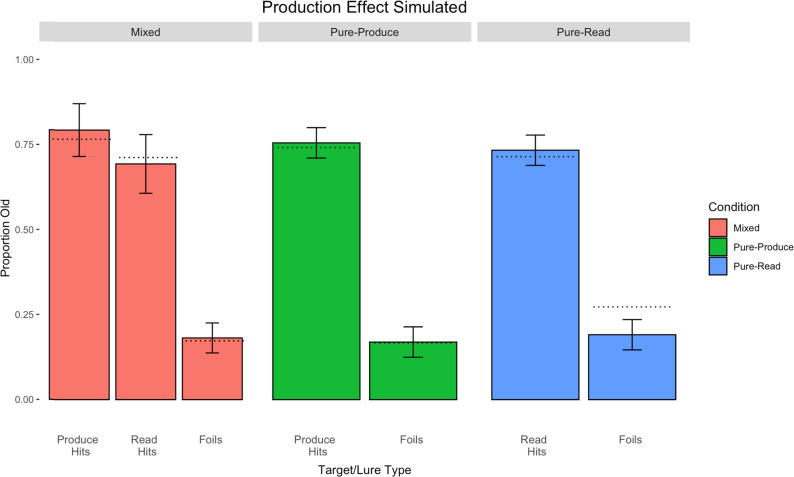
Simulation results of the production effect in Experiment 1 integrating strength and distinctiveness. Mixed-list parameters: *L* = 0.057 for produced targets, *L* = 0.057 for read targets, and the number of extra production features for produced targets was 250. The decision criterion was set to a slightly conservative value of 0.4625. Error bars represent standard deviations. Dotted lines represent corresponding empirical means.

Figure 5 shows the simulation results of the mixed-list condition on the left-hand side, the pure-produce condition in the middle, and the pure-read condition on the right. By assuming that produced targets are more strongly encoded than read targets and that produced targets were encoded with some extra distinctive information, we are able to generally reproduce the pattern of results that we obtained in Experiment 1 across the three conditions, RMSE = 0.0347.

However, the current simulation missed some key aspects of our data. First, the model in its current form predicts a larger mixed-list production effect than what we observed in our empirical data (a 10% vs. 6% production advantage). Second, the model also predicts a lower rate of false alarms to the foils in the pure-read condition. Although the model fit the data fairly well, we sought to explore whether we might get a better fit with only contributions of strength. We next present a simulation of Experiment 1 using only a strength mechanism, where no additional production features were added and where the iterative retrieval mechanism was omitted.

**Figure 6:**
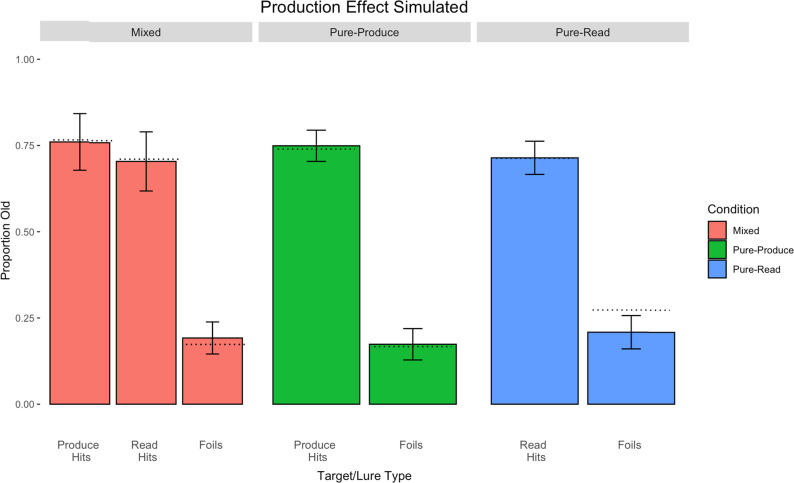
Simulation results of the production effect data in Experiment 1 assuming only strength. Mixed-list parameters: *L* = 0.0425 for produced targets and *L* = 0.0375 for read targets. There were no extra production features in the second simulation, as we did not assume any contributions of distinctiveness. The decision criterion was set to a slightly conservative value of 0.4625. Error bars represent standard deviations.

Figure 6 shows the simulation results of the mixed-list condition on the left-hand side, the pure-produce condition in the middle, and the pure-read condition on the right. By assuming that produced targets are more strongly encoded than read targets, we were able to closely reproduce the pattern of results that we obtained in Experiment 1 across the three conditions, RMSE = 0.0264.

Particularly, the model captured the production effect in the mixed-list condition and captured the trend of a pure-list production effect in the pure-list conditions. The rate of false alarms was different across the three different conditions, and the model was also able to capture this trend, although it underpredicted the heightened rate of false alarms in the pure-read condition. In the pure-read condition, participants had a tendency to say yes overall more often than in the pure-produce condition. However, for simplicity, we kept the decision criterion in our model at a slightly more conservative value, as we did in the following simulation of the directed forgetting experiment. Further, as seen in our empirical data, the model captures a muted *distinctiveness* pattern, where hits in both of the pure conditions sit between the hit rates in the mixed condition, even though there is no overt distinctiveness mechanism defined within the model. Moreover, we obtain a better model fit assuming only strength versus assuming strength and distinctiveness (RMSE = 0.0264 vs. 0.0347).

### Simulation of Experiment 2

Although our data again favored a strength mechanism in Experiment 2, we conducted the simulations for the directed forgetting procedure in the same way as the production effect procedure. Once again, we applied both the combined model integrating both strength and distinctiveness mechanisms, as well as the strength-based model of MINERVA 2 (assuming that R-cued items were encoded with more intact features than F-cued items).

**Figure 7:**
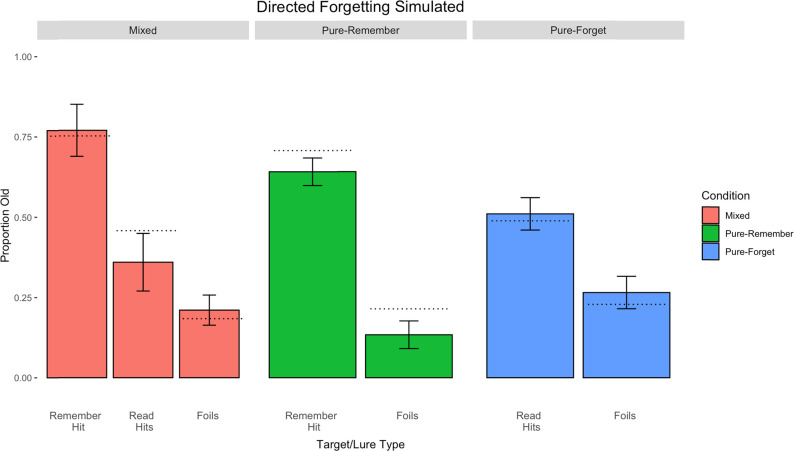
Simulation results of the directed forgetting data in Experiment 2. Parameters: *L* = 0.050 for R-cued targets and *L* = 0.026 for F-cued targets, and the number of extra elaborative features was 250. The decision criterion was set to a conservative value of 0.3875. Error bars represent standard deviations, and dotted lines represent corresponding empirical means.

Figure 7 shows the simulation results of the directed forgetting procedure, with the mixed-list condition on the left-hand side, the pure-remember condition in the middle, and the pure-forget condition on the right. By assuming that R-cued targets are more strongly encoded than F-cued targets and that there are extra features to account for the elaborative processing of a *remember* instruction, we can roughly capture the pattern of results observed in Experiment 2 across the three conditions, RMSE = 0.0578.

However, the simulation missed some key aspects of our data. The combined model integrating strength and distinctiveness predicted a lower hit rate for F-cued items in the mixed-list condition as well as the hit rate and false alarms in pure-remember condition.

As we observed signatures of strength in our directed forgetting data, we again sought to explore if the more parsimonious strength-based model could better capture the patterns in our data.

**Figure 8:**
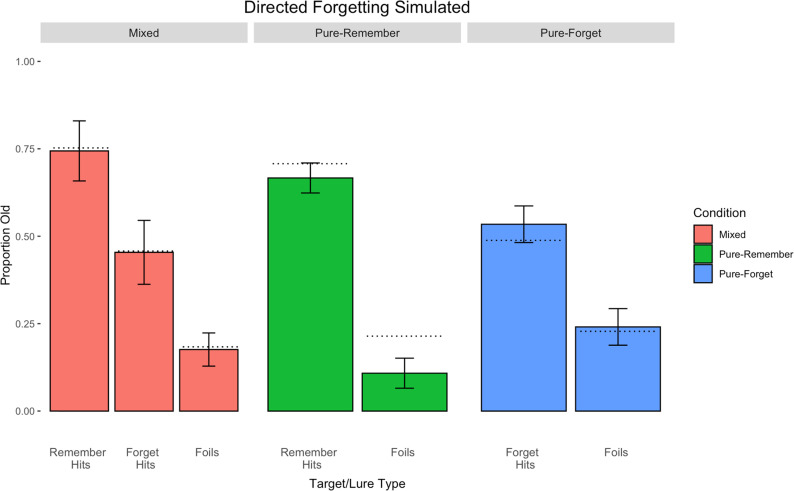
Simulation results of the directed forgetting data in Experiment 2. Parameters: *L* = 0.0425 for R-cued targets and *L* = 0.0226 for F-cued targets. The decision criterion was set to a conservative value of 0.3875. Error bars represent standard deviations, and dotted lines represent corresponding empirical means.

Figure 8 shows the simulation results of the directed forgetting procedure, with the mixed-list condition on the left-hand side, the pure-remember condition in the middle, and the pure-forget condition on the right. By assuming that R-cued targets are more strongly encoded than F-cued targets, we can closely replicate the pattern of results observed in Experiment 2 across the three conditions, RMSE = 0.0471.

As can be seen, the model reproduces the directed forgetting effects in both the mixed- and pure-list designs. In comparison to the production effect, we had to adopt a more conservative decision criterion, driven by the fact that participants gave far fewer yes decisions in the pure-forget condition than in the pure-read condition. Additionally, the model once again captured a muted distinctiveness pattern, where hits in both of the pure conditions sat between the hit rates in the mixed condition.

Notably, when comparing the parameter values across the production effect and directed forgetting simulations, the value of *L* for produced or R-cued targets remains constant. However, the value of *L* for read or F-cued targets varies across procedures to account for the size of the effect. Our empirical data corroborate this, showing a larger cost to F-cued targets compared to read targets, which yields a greater magnitude effect of directed forgetting relative to production.

### Simulation of Experiment 3

Although we found no statistical difference in the mixed-list condition in Experiment 3 compared to Experiment 1, when the cue to produce or read was presented either concurrently or shortly thereafter, we simulated the mixed-list results of Experiment 3 for a complete account of our data. As we obtained the best fit with the strength-based model for Experiment 1, we simulated Experiment 3 with this same model.

**Figure 9:**
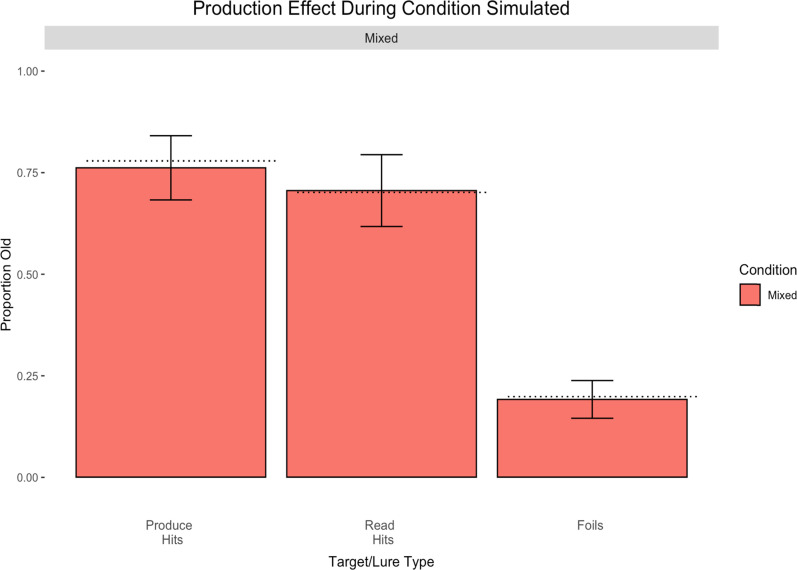
Simulation results of the mixed-list production effect data in Experiment 3. Parameters: *L* = 0.0425 for produce targets and *L* = 0.0375 for read targets. The decision criterion was set to a slightly conservative value of 0.4625, as in Experiment 1. Error bars represent standard deviations, and dotted lines represent corresponding empirical means.

Figure 9 shows the simulation results of the mixed-list production effect procedure when the cue to produce or read was presented concurrently with stimulus presentation. With a higher value of *L* for produce targets than read targets, we again capture the pattern or results as seen in Experiment 3, RMSE = 0.0477. Notably, we fit the Experiment 3 data well using the same parameters as used to simulate Experiment 1. This is corroborated by our empirical data, where we observed no difference when the cue to produce or read was presented concurrently with stimulus presentation or shortly thereafter.

## General Discussion

When given the instruction to *produce* or *remember*, in comparison to the instruction to *read* or *forget*, a stable increase in participants’ recognition memory performance can be observed for both classes of elaboratively encoded items. However, our data show that the magnitude of this benefit differs across tasks, which is primarily due to the varying costs associated with the second class of items (i.e., the read and forget items). Specifically, the directed forgetting procedure produces a larger cost to forget items than the production effect incurs to read items.

In our empirical data, we obtained a pure-list directed forgetting effect but failed to observe the elusive pure-list production effect. However, the pure-list difference was in the predicted direction, and as evidenced by previous examinations, this difference is known to be small (Fawcett, 2013; see also Fawcett et al., 2023). Furthermore, although the differences between hits in the pure-produce and pure-read conditions were small, participants had more false alarms in the pure-read condition, suggesting that their ability to discriminate targets from lures was weaker in the pure-read condition.

Three main hypotheses have been proposed to explain the item-method directed forgetting effect: selective rehearsal (Hourihan & Taylor, 2006; MacLeod, 1998; Woodward & Bjork, 1971), retrieval inhibition (Bjork, 1989; Brasden et al., 1993), and contextual change (Fawcett et al., 2024; Sahakyan & Kelley, 2002). These suggest that the effect could be due to (1) increased memory rehearsal for elaborately encoded items, (2) active suppression of F-cued items, or (3) a shift in internal context or mental state associated with two different classes of items. In the present investigation, we provide evidence that supports a strength mechanism as the primary driver of the directed forgetting effect, as demonstrated by our model. With the simplifying assumption that R-cued items are encoded with more intact features into memory than F-cued items, our model can closely capture the patterns we observed in our data, without the need for any additional rehearsal or inhibitory assumptions.

In this study, our goal was to further explore the role of strength and/or distinctiveness and to what extent these two principles contribute and explain our data in memory studies. Specifically, we investigated whether these principles could reconcile two notable memory phenomena: the directed forgetting effect and the production effect. When comparing mixed- versus pure-list designs to assess the contributions of strength and distinctiveness, we found no strong evidence in favor of distinctiveness in either procedure. However, we were able to best account for the results using a strength mechanism. Importantly, the strength-based model produced excellent fits to the empirical data from the directed forgetting and production effect tasks.

Typically, with spoken production, the interaction between production and experimental design (mixed vs. pure-list) is significant, lending support to a distinctiveness mechanism. In the current study, we do not believe the lack of a significant interaction between production-by-typing and experimental design to be due to different mechanisms underlying the two procedures (typing vs. spoken production), but rather due to the richness of the representation that is encoded into memory. If spoken production encodes both sensory feedback (auditory) and motoric features into memory, production-by-typing only encodes motoric features into memory, as there is no auditory component to this modality. As such, the differing pattern of results obtained from spoken production or production-by-typing suggests that production-by-typing is a shallower form of encoding in comparison, but robust, nonetheless. Moreover, the difference between pure- versus mixed-lists was in the right direction but did not reach the level of significance.

Rather than strength or distinctiveness, we think it is more likely that these two mechanisms exist on a continuum and can work together in tandem, where one mechanism can be favored over the other, depending on the task at hand. Therefore, we believe that future studies should examine under what circumstances a greater contribution of the distinctiveness mechanism is needed to accommodate findings compared to strength and under what circumstances each mechanism adds predictive power to the model.

Assuming a strength mechanism, more intact features for elaboratively encoded items result in a richer representation of this class of items. This aligns with the levels of processing explanation (Craik & Lockhart, 1972; Craik & Tulving, 1975). From this perspective, varying levels of strength can be equated to varying levels of processing. In particular, the differing magnitudes of each effect due to the performance of the 'read' or 'F-cued' items can be explained by varying levels of processing in the two cases.

One limitation of the current study is the inclusion of the pure-forget condition in the directed forgetting procedure. It could be argued that participants were completely disengaged from the task in this condition since all words were to be forgotten. However, participants were not informed of the specific procedure they were assigned to. They were told that, depending on their assigned condition, they might be instructed to remember all the words or forget all the words. Therefore, we are hesitant to conclude that participants disengaged entirely, as they may have been waiting for an R-cued word, at least for part of the time.

It may also be argued that our findings in the pure-forget condition (and other conditions including an F-cue instruction) reflect a demand characteristic, such that participants are trying to behave as a *good* participant, by responding that they do not recognize an F-cued item (as they were instructed to forget it), even though they may correctly recognize it from the study phase. To address the issue in directed forgetting studies, a tactic that has been used is monetary compensation for each correctly recognized item, regardless of the cue type. However, when employing such a tactic, investigations consistently show that the forgetting effect remains, even when there is an incentive to remember the F-cued items (Aguirre et al., 2020; Bjork & Woodward, 1973; Geiselman et al., 1985; MacLeod, 1999; Woodward & Bjork, 1971). Although we did not employ such a tactic in our investigation, we believe that the evidence in these demonstrations presents a strong and compelling argument against the claim that a demand characteristic might be driving our pattern of findings. MacLeod (1999) also investigated the role of demand characteristics in directed forgetting and found that under both list and item methods, offering monetary compensation for recall or recognition of F-cued items did not result in participants having any better performance for items that were to-be-forgotten.

Similarly, a pure-read condition in the production effect might invite participants to relax or disengage, as no action is required from them, particularly when compared to mixed-list or pure-produce conditions. Thus, both pure-read and pure-forget conditions seem unnatural when compared to mixed-list or pure-remember/read conditions. However, these conditions were included to complete the full design for assessing strength and/or distinctiveness in the two cases.

In most study instruction manipulations, we rely on trusting that participants are following our instructions, especially when there are no overt measures to collect (e.g., asking participants to imagine doing a task or to remember or forget words). The evidence for participant compliance lies within the data. Our data suggest that participants treat pure-forget and pure-read items differently compared to pure-remember or mixed-list items, as evidenced by differing hit and false alarm rates. Nevertheless, the purpose of including the pure-list conditions in this instance was to chiefly assess strength and/or distinctiveness.

Although the production effect tends to be larger with spoken production over typed production, the current results with typing show that the effect remains robust. The difference in magnitude between spoken versus typed production we do not believe to be calling upon different processes, but rather is due to the quality of the signal that is emitted. For example,Murray (1965a, 1965b; Murray et al., 1974) found that the magnitude of recognition performance increased incrementally when the modality of production moved from silent to whisper to aloud. Similarly, findings from related work on the drawing effect also demonstrate that there is a larger effect when words are drawn versus when they are viewed or written (Fernandes et al., 2018). Similar to the production effect, it is argued that the mechanism behind the drawing effect involves elaborative, motor, and pictorial components of a memory trace. However, there have been discrepant findings in relation to this claim when the modality of production is by singing. Whitridge et al. (2024) found that the production effect is not always larger for singing than saying words aloud, particularly when words do not appear in the same color at study and at test.

Moreover, according to Caplan and Guitard (2024), it could be that manual typing production places an additional emphasis on motor and orthographic features, which would exist in a higher density subspace than other modalities of production where these might exist in a subspace that includes motor, orthographic, and phonological features. Finally, in terms the differing magnitudes of production effects found across papers, the nature of the stimuli might play an important role, such that differences in frequency, word length, presentation rate, etc., could affect the results.

### The Current Model

A careful reader may wonder why we chose to use real representations drawn from a normal distribution with *M* = 0 and *SD* = 1/√(*N*_dim_) instead of discrete binary values as typically used in a classical MINERVA 2 approach (−1’s and +1’s). Going forward, we hope to adopt more structured representations, such as those derived from natural language processing models (e.g., LSA; Landauer & Dumais, 1997). By using real-valued representations and, consequently, a cosine similarity calculation, going forward, the model is equipped to deal with these kinds of representations. All these changes are forward-looking.

In the same vein, a limitation of the current modeling approach is the random representations of words in memory. By representing words in this fashion, it assumes that all words in memory are orthogonal. However, in practice, words are not orthogonal and share similarities in dimensions such as semantics, orthography, and phonology. Thus, future work should aim to address this issue, where more structured representations could be used.

Although previous to this there are two separate mechanisms of the production effect in the MINERVA 2 approach, it is important to note that the difference between the two is not a large one. The *strength-based* mechanism of the production effect accounts for the typical pattern of results by assuming that there are more intact features in memory (i.e., a richer representation) for produced items versus read items. The *distinctiveness-based* mechanism of the production effect accounts for the typical pattern of results by assuming that there are additional features added to memory for produced items versus read items (again, a richer representation). The critical difference between the two models is the iterative retrieval mechanism that operates in the distinctiveness-based model. Jamieson et al. (2016) accept this and note that “if distinctiveness and strength both work by adding features to a trace in memory, they are correlated concepts…” (p. 160). Thus, although one may call one implementation of the model a *strength* mechanism, and the other a *distinctiveness* mechanism, the two models are very close to being mathematically equivalent. Therefore, formal mathematical models can serve as valuable tools to overcome the limitations of vague verbal descriptions of memory effects.

In addition, this notion of creating a richer representation in memory is amenable to other recent models of the production effect (see Caplan & Guitard, 2024). In this approach, the authors vary the dimensionality and sparsity of vector subspaces, which is akin to a strength-based mechanism in the MINERVA 2 approach (i.e., the number of non-zero features stored in memory).

It should be noted that although the current strength-based model best accounts for the results of the experiments that are presented in this paper, it may not account for all data obtained from other production effect experiments, most of which confirm that the production effect arises from distinctiveness rather than strength. Thus, going forward, it would be beneficial to test the model where strength and distinctiveness mechanisms are combined and varied to account for the data in the full database of experimental effects. Critically, although the version of the model that combines strength and distinctiveness does not best fit our current data, we know it could serve as a valuable tool to assess most other production experiments where larger contributions of distinctiveness are observed compared to strength. As such, we find this to be the stronger model of the two presented in this paper.

### Conclusion

In our study, we investigated the effects of production and directed forgetting on recognition using both mixed-list and pure-list designs. Our findings reveal that the two effects, commonly attributed to some form of elaborative processing, exhibit variations in effect size. We provide deeper insights into two theoretical mechanisms, strength, and distinctiveness that drive the effects of elaborative processing, whereby distinctiveness need not always be assumed within formal models.

Our findings also provide valuable insights for modeling efforts of directed forgetting and the production effect. Our pattern of results highlights the need for further exploration and refinement of theories regarding the production effect, underscoring different scenarios where strength and distinctiveness may work in concert rather than compete for control in a mutually exclusive arena. Additionally, our research goes beyond advancing our comprehension of directed forgetting and the production effect; it also deepens our understanding of the underlying mechanisms at play. Our data and model suggest that the dichotomy between strength and distinctiveness may oversimplify the matter, as these processes likely interact, and are correlated mechanisms. A comprehensive account must acknowledge this complexity to further delineate the contributions of strength and/or distinctiveness in effects of elaborative processing.

## Notes

**Publication Ethics:** All participants of Experiments 1–3 provided informed consent.

**Open Science:** To the best of my ability and knowledge, I have provided all original materials and clear references to all other materials via a stable online repository.

Open Data: Data are available from https://osf.io/sbfwm/ (Spear et al., 2024).

Open Materials: Materials are available from https://osf.io/sbfwm/ (Spear et al., 2024).

Open Code: The code is available from https://osf.io/sbfwm/ (Spear et al., 2024).

Preregistration: My manuscript contains no experiment with a completely executed preregistration.

## Appendix

**Table A1:** 120 words taken from MacDonald and MacLeod (1998)

Stimuli					
ACCOUNT	CENTURY	GARDEN	LANGUAGE	PLATE	TEACHER
ADDRESS	CLOTHES	GLASS	LAUGH	POCKET	THEATER
AFTERNOON	DAUGHTER	GRAVITY	LEATHER	PORCH	THREAD
AMOUNT	DEBATE	GUARDIAN	LESSON	POWDER	TICKET
ANSWER	DEPARTMENT	HANDLE	MACHINE	QUARREL	TRAFFIC
ARROW	DINNER	HARBOR	MARKET	QUARTER	TRAVEL
ATTENTION	DIRECTION	HISTORY	MEADOW	QUEEN	TREASURE
ATTITUDE	DISTANCE	HOLIDAY	MERCHANT	RECORD	TROUSERS
AUTHOR	EDUCATION	INDUSTRY	MESSAGE	RESORT	TURNIP
AVENUE	ELECTION	INVENTION	MINUTE	REWARD	UNCLE
BASKET	ENGINE	INVITATION	NEIGHBOR	RIVER	UNIFORM
BATTERY	ENTRANCE	ISLAND	NEPHEW	SAILOR	VACATION
BEAUTY	ENVELOPE	JOURNEY	OCEAN	SCHOOL	VALLEY
BORDER	EVENING	JUDGE	OFFICE	SHADOW	VICTORY
BRANCH	FACTORY	JUSTICE	ORCHARD	SHOULDER	VILLAGE
BUILDING	FASHION	KETTLE	PACKAGE	SPEECH	WAGON
CAMPAIGN	FOREST	KINGDOM	PAINTING	STATION	WHEAT
CAPITAL	FOUNDATION	KITCHEN	PARTNER	STEAM	WHEEL
CAPTAIN	FRIEND	KNOCK	PEACE	STREAM	WHISPER
CASTLE	FURNITURE	LADDER	PEBBLE	SUMMER	WINTER
